# Pharmacy engagement in TB prevention and care: not if, but how?

**DOI:** 10.1136/bmjgh-2023-013104

**Published:** 2023-07-20

**Authors:** Rosalind Miller, Francis Wafula, Kinz ul Eman, PS Rakesh, Bolanle Olusola Faleye, Catherine Duggan, Gonçalo Sousa Pinto, Petra Heitkamp, Namrata Rana, Joel Shyam Klinton, Giorgia Sulis, Charity Oga-Omenka, Madhukar Pai

**Affiliations:** 1 TBPPM Learning Network, Research Institute McGill University Health Center (RI-MUHC), Montreal, Quebec, Canada; 2 Institute of Healthcare Management, Strathmore University Strathmore Business School, Nairobi, Kenya; 3 Dopasi Foundation, Islamabad, Pakistan; 4 Amity Institute of Public Health & Hospital Administration, Amity University, Noida, Uttar Pradesh, India; 5 USAID Local Health Systems Sustainability project (LHSS), Abt Associates Nigeria, Lagos, Nigeria; 6 International Pharmaceutical Federation (FIP), The Hague, The Netherlands; 7 School of Epidemiology and Public Health, University of Ottawa, Ottawa, Ontario, Canada; 8 Clinical Epidemiology Program, Ottawa Hospital Research Institute, Ottawa, Ontario, Canada; 9 School of Public Health Sciences, University of Waterloo, Waterloo, Ontario, Canada; 10 McGill School of Population and Global Health, McGill University, Montreal, Quebec, Canada

**Keywords:** Tuberculosis, Pharmacology, Health services research, Health education and promotion

## Introduction

Over four million people with tuberculosis (TB) globally (45% of the estimated TB burden) are ‘missing’.^1^ This gap of not-reported people with TB is widening, up from 2.1 million in 2019 to 4.1 million in 2021 largely due to the COVID-19 pandemic.^1^

Most individuals with TB seek initial care at the level of primary care, and community pharmacies are a common first point of contact for people with symptoms suggestive of TB such as long-lasting cough and fever, making them well placed to find people with TB in high-burden settings.^2–5^ The COVID-19 pandemic showcased how effectively pharmacists and other pharmacy professionals can be engaged and leveraged to provide information, point-of-care (POC) testing and vaccination.^6 7^The role of pharmacists in TB care was also described and supported in a joint statement by the World Health Organization (WHO) and the International Pharmaceutical Federation (FIP) in 2011.^8^

Prior research has identified inadequacies in how pharmacy professionals manage people with presumptive TB, including inappropriate over-the-counter sales of medicines such as fluoroquinolone antibiotics, steroids and cough suppressants which can mask the symptoms of TB. Referral for sputum testing is also noticeably lacking.^9 10^

These shortfalls in quality of management have been mostly attributed to three areas: (1) low TB knowledge among pharmacy staff, (2) inadequate systems for referring clients for testing or notifying TB (such as lack of digital reporting systems) and (3) lack of support, such as training or appropriate funding models.^11 12^ Evidence suggests that engaging private providers can contribute to quality TB care,^12 13^ improve TB diagnosis,^13^ expand case detection at roughly the same cost as the public sector.^14^ As pharmacies are often the first point of access to care, the question of whether pharmacies should be engaged in identifying people with TB is a rhetorical one. The dialogue must shift towards how to engage pharmacies more effectively and address the bottlenecks.^15^ Here, we propose three priority actions that should be taken, especially in high-burden settings:

## Access to clear and concise pharmacy-specific guidance, backed by national or regional pharmacy associations, for the management of people presenting with cough

**Figure 1:**
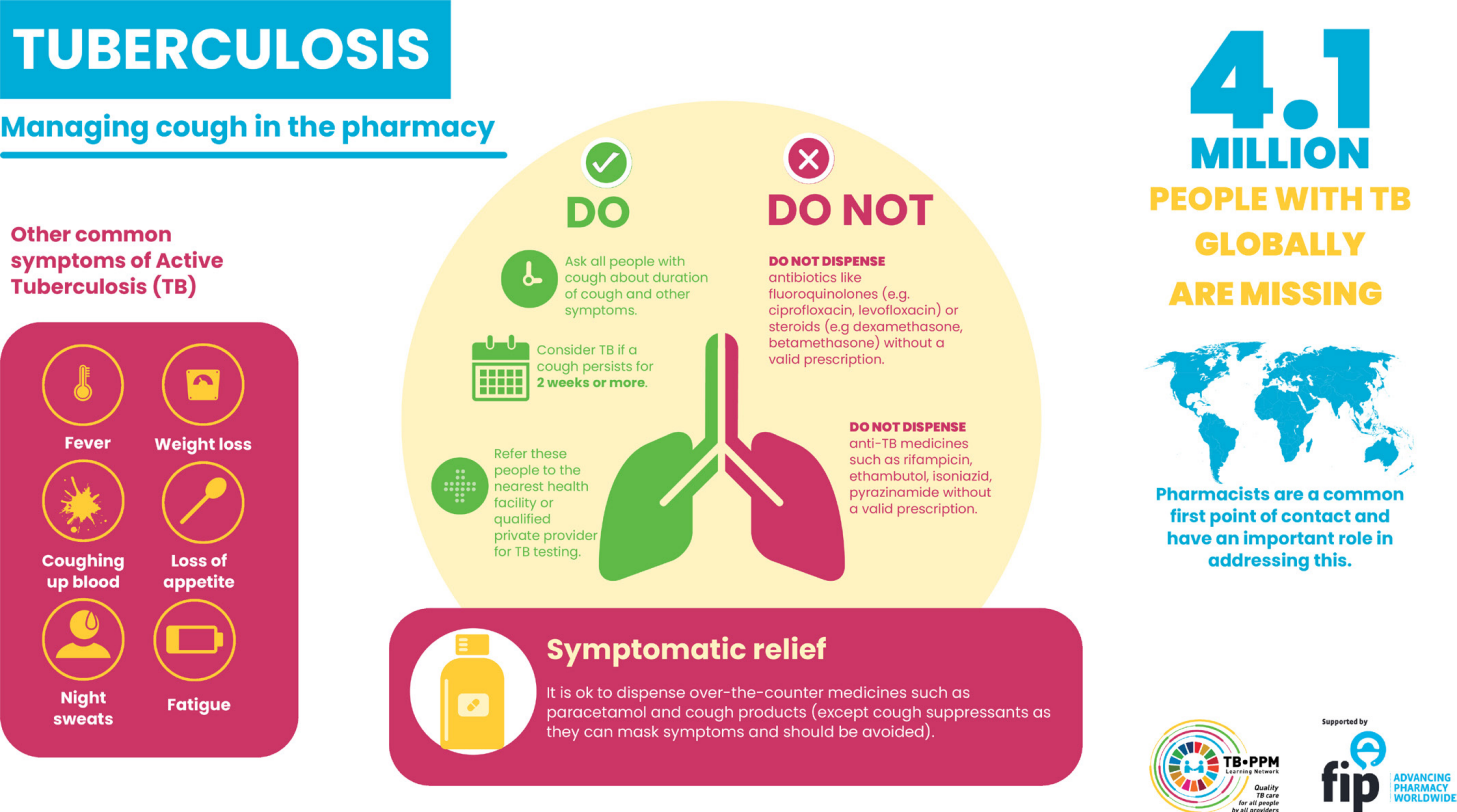
Infographic for managing cough and other tuberculosis (TB)-presumptive symptoms in the pharmacy. Coordinated by the TB-PPM Learning Network.

To date, there is little specific guidance or practical tools available on TB management for pharmacists, codesigned with pharmacy professionals or societies.^12^ To address this gap, we set about creating a simple, easy to understand and evidence-based infographic to use as an educational tool (figure 1), providing pharmacies with key information, as well as ‘do’s’ and ‘do not’s’, to refer to when they encounter people presenting with cough. The content of the infographic was created, debated and revised through extensive stakeholder engagement, including academics, programme implementers and representatives from international organisations and national TB programmes (NTPs), and is in line with international TB standards.^12 16^ A survey among stakeholders provided further input and feedback on an initial draft, which was later presented, discussed and subsequently revised. The infographic is supported by the International Pharmaceutical Federation (FIP) - the global organisation representing pharmacists, pharmaceutical scientists and pharmaceutical scientists and educators - and stakeholders in 14 high-TB burden countries (Bangladesh, Ghana, India, Indonesia, Kenya, Myanmar, Nepal, Nigeria, Pakistan, Philippines, South-Africa, Tanzania, Viet Nam, Zambia) implementing a variety of pharmacy engagement models. In consultation with NTPs, national pharmacists associations and key implementing partners, the tool can be adapted, adopted, translated into local languages and disseminated in a range of settings. The infographic is currently being disseminated in Zambia to 400 pharmacies, and the 13 other countries have been contacted regarding adapting and dissemination in line with their national policy and strategies. (Contact the corresponding author for use and adaptation of the infographic, and to include national logos).

The aforementioned infographic aims to aid early TB diagnosis by increasing awareness of symptoms and global burden. Moving forward, we suggest implementing more targeted and context-specific educational tools to better equip pharmacy professionals address the key challenges of over-the-counter dispensing of anti-TB medications; limited knowledge of symptoms and management; inadequate referral processes between pharmacies and testing centres and the need for incentives.

## Establishment of structured mechanisms for pharmacies to support people with presumptive TB

Drawing on global experiences of interventions for improving engagement of pharmacists in TB care, Bigio *et al* highlight the need for establishing and improving mechanisms that allow people with presumptive TB to be screened in pharmacies and effectively linked to national TB systems.^11^ Typically, substantial loss to follow-up happens at the stage of referring people for TB testing. Reports indicate that this may persist, even when efforts are introduced to mitigate the loss (eg, providing free transport).^11^ Further, NTPs may not be adequately engaging pharmacies in the continuum of care for people with presumptive TB.^17^ Establishing POC testing in the pharmacy is an obvious way to prevent such loss and has the potential to reduce diagnostic delays. Although there is no simple POC test that can be used by pharmacists, other screening approaches have been tried. In India, digital chest X-ray (CXR) vouchers were given by pharmacists to people with presumptive TB resulting in substantial increases in case detection.^13^ Programmes in countries such as Kenya and Nigeria have attempted to address this challenge by introducing symptom screening for TB, initiated at pharmacists through questionnaires or mobile apps.^11 18^ This approach paves the way for a new future of effective pharmacist engagement. For example, Nigeria is implementing a ‘hub-and-spoke’ model, where Patent Medicine Vendors (spokes) drive patient traffic to government or recognised private laboratories (hubs) by screening for TB and collecting sputum samples.^18 19^ Referred patients benefit from Xpert MTB/Rif testing, that is, a rapid molecular test for detecting TB and rifampicin-resistant TB, which can be used at the POC. Alternatively, or in addition, they refer people with presumptive TB to medical facilities for supplementary assessment or treatment. Furthermore, public sector TB clinics could engage pharmacies as community partners to provide local support to people with TB by monitoring their treatment adherence and offering counselling support in between monthly or bimonthly appointments.^20^ As such, pharmacists provide patient counselling and support, adherence monitoring and adverse events’ reporting (pharmacovigilance). While this model shows promise, research needs to confirm this evidence, and concerns persist over sustainability in the face of limited health system support.

## Use of advancements in digital technology to improve the linkage between pharmacies and TB programs and surveillance

While paper-based forms and printed referral slips have been used in the past, there is an increased recognition of the importance of embracing digital technologies such as digital linkage and notification and development of dashboards to improve decision-making based on data collected from the pharmacies.^21^

In Pakistan, a novel app (e-TB) has shown initial success in improving case notification.^18^ The social enterprise initiative was started by the DOPASI Foundation to support the order for mandatory notification. Pharmacists sign up using licence numbers, then report via the app every time they receive a prescription for anti-TB medications, providing patient and prescriber details and an uploaded photo of the prescription. Patients’ telephone number is then used for follow-up with messages to support the patient during treatment and improve adherence. Around 16 000 TB notifications were made in a year between April 2021 and end of March 2022 from 2981 pharmacies (representing a 20%–30% increase in TB notifications per district). In India, the Nikshay portal allows for electronic TB notification and data analysis, including for pharmacists connected with the NTP. Further, a pilot initiative is underway to capture relevant patient, provider and anti-TB medication data and integrate a billing software to generate real-time information.^22^ In South-East Asia, a digital networking app called ‘SwipeRx’ has been shown to facilitate and educate pharmacy professionals increasing referrals for TB screening, as illustrated in Viet Nam^23^ and Indonesia.^23^ Other countries could emulate such models to enable referral for testing and other services.

E-pharmacy is a growing industry that has the potential to revolutionise the way people access healthcare services, including the delivery of TB drugs and testing. With the widespread availability of the internet and the increasing adoption of digital technologies, e-pharmacies have become an attractive option for patients who want to order their medications online and have them delivered to their homes.^24 25^

Design of mobile technology to improve linkages between pharmacies and TB programmes must ensure that they include two-way feedback/communication between the government and the pharmacy. Pharmacists will be more readily engaged if they receive regular feedback on referred patients, especially those leading to a positive TB test.

## Conclusion

The historic role of pharmacists in TB care and prevention has centred on dispensing anti-TB medications; however, it is clear that they can assume a larger role. We have outlined three key actions that could harness the potential of community pharmacists in supporting the efforts to End TB. The pharmacy-specific infographic which we have produced collaboratively, serves as an important first step towards ensuring pharmacies have access to evidence-based management advice. There is a need for more detailed guidance as well as expansion of digital systems to enable reporting and referral of people who might have TB. Additionally, early engagement of pharmacists in collecting sputum linked to labs, referrals for CXR with free vouchers or referral for TB testing at close by sites, serves as a component of health system preparedness for roll-out of POC diagnostics and TB vaccines that are in the research pipeline. As described above, there is plentiful scope to expand the role of the pharmacist further to allow for in-pharmacy TB testing, diagnosis and notification. These initiatives require strong partnerships between TB programmes, national association of pharmacists and allied cadres. Policy should consider introducing appropriate and sustainable funding models to capture the missing people with TB via pharmacies.

## Data Availability

All data relevant to the study are included in the article.

## References

[1] World health organization. Global tuberculosis report Geneva World Health Organization; 2022.

[2] Mistry N, Rangan S, Dholakia Y, et al. Correction: durations and delays in care seeking, diagnosis and treatment initiation in uncomplicated pulmonary tuberculosis patients in Mumbai, India. PLoS One 2016;11:e0160796. 10.1371/journal.pone.0160796 27486896 PMC4972312

[3] Miller R, Goodman C. Performance of retail pharmacies in low- and middle-income Asian settings: a systematic review. Health Policy Plan 2016;31:940–53.:czw007. 10.1093/heapol/czw007 26962123 PMC4977427

[4] Surya A, Setyaningsih B, Suryani Nasution H, et al. Quality tuberculosis care in Indonesia: using patient pathway analysis to optimize public-private collaboration. J Infect Dis 2017;216(suppl_7):S724–32. 10.1093/infdis/jix379 29117347 PMC5853837

[5] Oga-Omenka C, Okafor U, Sulis G. Engaigng pharmacists and medicine vendors in antimicrobial stewardship in Lmics. Lancet Infect Dis 2023;23:786–7. 10.1016/S1473-3099(23)00342-0 37209708

[6] Mbunge E, Batani J, Gaobotse G, et al. Virtual Healthcare services and digital health technologies deployed during coronavirus disease 2019 (COVID-19) pandemic in South Africa: a systematic review. Glob Health J 2022;6:102–13. 10.1016/j.glohj.2022.03.001 35282399 PMC8897959

[7] Klinton JS, Heitkamp P, Rashid A, et al. One year of COVID-19 and its impact on private provider engagement for TB: a rapid assessment of intermediary NGOs in seven high TB burden countries. J Clin Tuberc Other Mycobact Dis 2021;25:100277. 10.1016/j.jctube.2021.100277 34545343 PMC8444472

[8] World Health Organization (WHO), International pharmaceutical Federation (FIP). The role of pharmacists in TB care was also described and supported in a joint statement by the world health Organization (WHO) and the International pharmaceutical Federation (FIP) in 2011. 2011. Available: https://www.who.int/news/item/05-09-2011-signing-of-a-new-tuberculosis-initiative-between-the-world-health-organization-and-the-international-pharmaceutical-federation

[9] Svadzian A, Daniels B, Sulis G, et al. Do private providers initiate anti-tuberculosis therapy on the basis of chest radiographs? A standardised patient study in urban India. Lancet Reg Health Southeast Asia 2023;13:100152. 10.1016/j.lansea.2023.100152 37383564 PMC10306035

[10] Satyanarayana S, Kwan A, Daniels B, et al. Use of standardised patients to assess antibiotic dispensing for tuberculosis by pharmacies in urban India: a cross-sectional study. Lancet Infect Dis 2016;16:1261–8. 10.1016/S1473-3099(16)30215-8 27568359 PMC5067371

[11] Bigio J, Aquilera Vasquez N, Huria L, et al. Engaging pharmacies in tuberculosis control: operational lessons from 19 case detection interventions in high-burden countries. BMJ Glob Health 2022;7:e008661. 10.1136/bmjgh-2022-008661 PMC9020292 35440442

[12] Miller R, Goodman C. Quality of tuberculosis care by pharmacies in low- and middle-income countries: gaps and opportunities. J Clin Tuberc Other Mycobact Dis 2020;18:100135. 10.1016/j.jctube.2019.100135 31872080 PMC6911950

[13] Daftary A, Satyanarayana S, Jha N, et al. Can community pharmacists improve tuberculosis case finding? A mixed methods intervention study in India. BMJ Glob Health 2019;4:e001417. 10.1136/bmjgh-2019-001417 PMC6528751 31179037

[14] Deo S, Jindal P, Gupta D, et al. What would it cost to scale-up private sector engagement efforts for tuberculosis care? Evidence from three pilot programs in India. PLoS One 2019;14:e0214928. 10.1371/journal.pone.0214928 31166942 PMC6550378

[15] Pradipta IS, Yanuar EO, Nurhijriah CY, et al. Practical models of pharmaceutical care for improving tuberculosis patient detection and treatment outcomes: a systematic Scoping review. Trop Med Infect Dis 2023;8:287. 10.3390/tropicalmed8050287 37235335 PMC10224363

[16] World Health Organization. Module 3: diagnosis-rapid diagnostics for tuberculosis detection. In: WHO consolidated guidelines on tuberculosis. World Health Organization, 2020. 33999549

[17] Menberu M, Kar S, Ranjan Behera M. Review on public private mix TB control strategy in India. Indian J Tuberc 2022;69:277–81. 10.1016/j.ijtb.2021.07.007 35760477

[18] TBPPM. Engaging pharmacies to end TB. 2022. Available: https://www.tbppm.org/networks/events/85012 [Accessed 16 Nov 2022].

[19] Ali T, Singh U, Ohikhuai C, et al. Partnering with the private laboratories to strengthen TB diagnostics in Nigeria. J Clin Tuberc Other Mycobact Dis 2023;31:100369. 10.1016/j.jctube.2023.100369 37122613 PMC10130621

[20] Daftary A, Jha N, Pai M. Enhancing the role of pharmacists in the cascade of tuberculosis care. J Epidemiol Glob Health 2017;7:1–4. 10.1016/j.jegh.2016.05.001 27260385 PMC7320509

[21] World Health Organization. WHO consultation on enhanced TB-PPM Dashboards with seven priority countries. 2022. Available: https://www.who.int/news/item/09-11-2022-global-meeting-on-strengthening-public-private-provider-engagement-calls-for-greater-collaboration-with-all-care-providers-to-get-the-tb-response-back-on-track-and-to-enhance-accountability [Accessed 16 Nov 2022].

[22] Gandhi R, Deepak KG, Verma G, et al. Engaging private pharmacies to help end TB in India. Int J Tuberc Lung Dis 2022;26:457–9. 10.5588/ijtld.21.0682 35505482 PMC9067430

[23] Tran PMT, Dam TA, Huynh HB, et al. Evaluating novel engagement mechanisms, yields and acceptability of tuberculosis screening at retail pharmacies in Ho Chi Minh city, Viet Nam. PLOS Glob Public Health 2022;2:e0000257. 10.1371/journal.pgph.0000257 36962503 PMC10021543

[24] Miller R, Wafula F, Onoka CA, et al. When technology precedes regulation: the challenges and opportunities of E-pharmacy in low-income and middle-income countries. BMJ Glob Health 2021;6:e005405. 10.1136/bmjgh-2021-005405 PMC8141442 34016578

[25] SHOPS Plus India. Active compliance and treatment strategy to tackle tuberculosis; 2020.

